# Coping mechanisms to drug stock-outs among patients seeking mental healthcare at outpatient department in Butabika National Referral Mental Hospital, Uganda: A cross-sectional study

**DOI:** 10.1371/journal.pone.0340898

**Published:** 2026-02-04

**Authors:** Angela Apio, Grifine Agarukamu, Gloria Nicole Amuron, Silas Ojuka, Mike Mugisha, Kalidi Rajab

**Affiliations:** 1 Department of Pharmacy, Makerere University, Kampala, Uganda; 2 Department of Pharmacy, Uganda Heart Institute, Kampala, Uganda; National University, SUDAN

## Abstract

**Background:**

Mental disorders are responsible for a significant proportion of global health burden especially in developing countries. In sub-Saharan Africa optimum care for mental health patients is constrained by frequent drug stock-outs. Patients who are victims of drug stock-outs are compelled to seek coping mechanisms to this challenge. These coping mechanisms may either be adaptive or maladaptive. Consequently, persons living with mental illnesses are prone to experiencing undesirable outcomes. This study purposed to explore coping mechanisms to drug stock-outs among patients seeking care at an outpatient department (OPD) of a national mental healthcare facility in Kampala, Uganda.

**Methods:**

This was an observational cross-sectional study. A sample size of 385 was obtained by systematic random sampling. Data was collected using a checklist and a questionnaire. The data was analyzed using SPSS version 29. Numerical variables were presented with means and standard deviations. Categorical variables were presented as frequencies and percentages. The results of the study were presented in tables, pie-charts and graphs.

**Results:**

Less than half of the participants; 164/385(42.60%), had their prescriptions fully filled with an average prescription fulfillment rate of 66.36%. Majority of the patients were victims of drug stock-outs. The most popular coping mechanisms were; out-of-pocket purchase of prescribed drugs from pharmacies, patients returning to hospital on a later date, skipping drug doses and using unprescribed herbal remedies. The commonest undesirable outcomes of coping mechanisms were; worsening of symptoms, insomnia and relapse of signs and symptoms.

**Conclusion:**

Drug stock-outs could have been responsible for low prescription fulfillment rates. This most likely prompted numerous patients to resort to alternative treatment modalities. These unprescribed treatment modalities could jeopardize patient prognosis and overall safety.

## Background

Mental health can be defined as a state of well-being that enables people to manage life’s typical challenges and perform effectively [1]. Globally the disability-adjusted life years (DALYs) associated with mental disorders increased from USD 80.8million in 1990 to USD 125.3million in 2019 [2]. However, a report on the global burden of diseases in 2019 indicated that mental disorders were responsible for 418 million DALYs which is 16% of global DALYs [3]. This has made mental illness one of the top ten causes of disease burden worldwide [2] resulting in significant loss of financial resources [4] and productivity [5].

The prevalence of mental disorders in Uganda has been estimated to be 22.9% in children and 24.2% in adults [6]. In 2018, up to 63% of all first time admissions in Butabika National Referral Mental Hospital(BNRMH) were psychosis related [7]. Depression alone was reported by a systematic review and a meta-analysis in Uganda to have a pooled prevalence of 30.2% [8]. Anxiety disorders have been reported to have a prevalence of 22.2% among adults in Uganda [6]. Combined together, depression and anxiety disorders affect approximately one in four persons in Uganda [6]. Despite this burden there is generally inadequate resource allocation towards mental healthcare in Uganda [9]. This ultimately affects adherence to prescribed mental health drugs. Approximately 36% of countries in sub-Saharan Africa spend less than 2% of their national health budgets on mental healthcare. This is significantly less than 12% that is recommended by the World Health Organization(WHO) [10].

Adherence to prescribed treatment is also impeded by numerous factors that include; ignorance, among patients, about mental illnesses [11] and fear of stigma and discrimination associated with mental illness [11–13]. Co-morbid conditions may complicate diagnosis and treatment of mental disorders [14–16]. These create the need for long term use of multiple drugs which consequently affects patient adherence to prescribed treatment [17,18]. Other factors that may hinder adherence to treatment among mental health patients are burden of adverse drug effects and high cost of drugs [19]. Furthermore, shortage of mental health professionals [20], especially in rural areas may cause; long waiting times for patients, delays in diagnosis and treatment, inconsistent care and poor coordination between primary care providers and mental health specialists. This discourages patients from seeking appropriate mental health care.

In cases of drug stock-outs patients and caregivers may resort to various coping mechanisms. These may be either adaptive or maladaptive. Adaptive coping mechanisms such as out-of-pocket purchase of drugs was also observed in Ethiopia where patients would use their personal savings to pay for medical expenses [21]. Others may resort to less expensive herbal treatments as observed among diabetic and hypertensive patients in the Ugandan districts of Mukono and Buikwe [22]. Patients may also adopt maladaptive coping mechanisms such as consulting traditional healers or seeking divine intervention as an alternative [23]. However, coping mechanisms to drug stock-outs may occasionally be hazardous to the patient or ineffective.

Some coping mechanisms expose patients to certain health risks that are important to note. These risks may include; use of counterfeit, substandard or illegal drugs which may be harmful to patients [24,25]. Patients may face relapse of symptoms, worsening of illness, or development of complications due to ineffectiveness of some coping mechanisms  [26]. Adaptive mechanisms to drug stock-outs can be convenient to the patient for the short term but may not be sustainable. For instance, there is increased financial burden on patients which may cause patients to mistrust the healthcare system [25]. Patients expect prescribed drugs to be availed at public facilities at no cost but are dismayed to realize that it is not always the case. Additionally only few patients have the socioeconomic capability to procure drugs out-of-pocket. This leads to poor adherence and eventually poor treatment outcomes [26].

## Methods

### Study aim

This study purposed to explore various coping mechanisms for drug stock-outs among patients seeking mental healthcare at OPD in Butabika National Referral Mental Hospital, Uganda.

### Study design

This was an observational cross-sectional study that employed quantitative methods.

### Study setting

BNRMH is located in Kampala district, which is in central Uganda. It is located in Nakawa division, some 11 kilometers south-east of Kampala’s downtown. The hospital’s coordinates are roughly 32.6334° E longitude and 0.3094° N latitude. Although the hospital’s primary responsibility is to care for mentally ill patients, there was integration of mental health and primary health care as part of the health sector strategic plan project (HSSP 1999/2000). Currently, the hospital manages an outpatient department that serves approximately 350 patients daily in the mental health clinics. Other health care services provided are dental, HIV/AIDS, orthopedic, minor surgery, and maternity and pediatric care.

### Study population

The study targeted patients who were seeking mental healthcare in OPD of BNRMH.

### Eligibility criteria

#### Inclusion criteria.

- Only outpatients seeking mental healthcare were included.
- Only adult patients were interviewed (18 and above).

#### Exclusion criteria.

- Patients with compromised comprehension abilities who did not have a caregiver (compromised comprehension ability was confirmed by the attending psychiatrist).

### Sample size determination

The sample size was determined using Kish Leslie formula,1965;


$$\text{n}=\frac{{\text{Z}}^{\text{2}}\text{pq}}{{\text{e}}^{\text{2}}}$$

Where:

(n) is the sample size required.

(Z) is the Z-score that corresponds to the desired level of confidence. For instance, for 95% confidence level, the Z-score is 1.96.

(p) is the estimated proportion of the population of patients whose prescriptions are not fully dispensed. We used 50% due to unavailable literature on the proportion of patients that have their prescriptions fully dispensed.

(q) is the complement of (p) i.e., q = (1 – p).

(e) is the margin of error or (Which is assumed to be 5%).

p = 50% or 0.5

Z = (95% level of confidence level therefore 1.96)

e = 5% or 0.05

Sample size therefore (n) = Z^2^*p (1-p)/ e^2^

n = 1.96^2^ * (1-0.5)/ 0.05^2^

n = 385 patients.

The sample size was 385 patients.

### Sampling procedure

Systematic random sampling was used to obtain the sample size. A total of 385 participants were selected from OPD of BNRMH on eight days in the month of data collection. For each week in the month two days were randomly selected on which participants would be selected. On each selected day, 48 participants (385/8) were selected and interviewed.

A total of 350 patients are seen each day at the hospital OPD. Therefore, to obtain 48 participants on the selected data collection day a sampling interval of seven was chosen; (350/48) =7. Every seventh (7^th^) patient was then enrolled into the study.

### Data collection

A checklist and a questionnaire were used to collect data. The checklist was used to extract data from participants’ prescriptions forms. The data extracted included; diagnosis, prescribed drugs and status of prescription fulfillment. The questionnaire was used to interview all participants in the study to collect data on coping mechanisms to drug stock-outs. Coping mechanisms to drug stock-outs were categorized as adaptive and maladaptive. Adaptive coping mechanisms to drug stock-outs was defined as medically constructive and safe endeavors of a patient or caregiver to access prescribed drugs or alternative medical assistance when they missed drugs. Maladaptive coping mechanisms to drug stock-outs were defined as irrational or unsafe alternatives to prescribed drugs sought by a patient or caregiver as a result of drug stock-out. The participants were allowed to report on all the coping mechanisms that applied to them. The research assistant ticked against all the responses that applied to the participant.

### Data analysis

The data was analyzed using SPSS version 29. Numerical variables were presented as means and standard deviations while categorical variables were presented as frequencies and percentages. The results of the study were presented in tables, pie-charts and graphs.

### Ethics approval

Ethical approval was obtained from Makerere University School of Health Sciences Research and Ethics Committee and assigned reference number MAKSHSREC-2024-652. This study was conducted in compliance with the declaration of Helsinki.

### Consent to participate

Participants offered written consent before taking part in this study.

### Availability of data and materials

The datasets used and/or analyzed during the current study have been made fully available to the journal.

## Results

### Demographics and characteristics of participants

The mean age of the participants was 38.3 ± 13.9 SD. More than half of the participants were male; 213(55.3%), while majority were entrepreneurs by occupation; 102(41.30%). Most of the participants had been diagnosed with psychosis; 233(60.52%), whereas over three quarters made a monthly visit to the hospital; 291(75.58%) (Table 1).

**Table 1:** Demographics and clinical characteristics of respondents.

Category/variable/parameter		Frequency n = 385(%)
**Sex**	Male	213 (55.30)
	Female	172 (44.70)
**Occupation**	Business/Entrepreneurial	159 (41.30)
	Peasant	78 (20.26)
	Unemployed	63 (16.36)
	Civil servants	43 (11.17)
	Student	42 (10.91)
Disease condition***	Psychosis	233 (48.54)
	Seizure disorders	120 (25.00)
	Mood disorders	111 (23.13)
	Others*	16 (3.33)
**Frequency of clinic visits**	Monthly	291 (75.58)
	Bimonthly	46 (11.95)
	Quarterly	35 (9.09)
	Others**	13 (3.38)

Mean Age (years): 38.3 ± 13.9 SD

Others*: “anxiety disorders”, “dementia” and “substance use disorders”.

Others**: “when unwell” and “when drugs are done”.

Disease condition***: frequency out of 480; some patients had multiple disease conditions

### Proportion of prescription fulfilment

**Fig 1:**
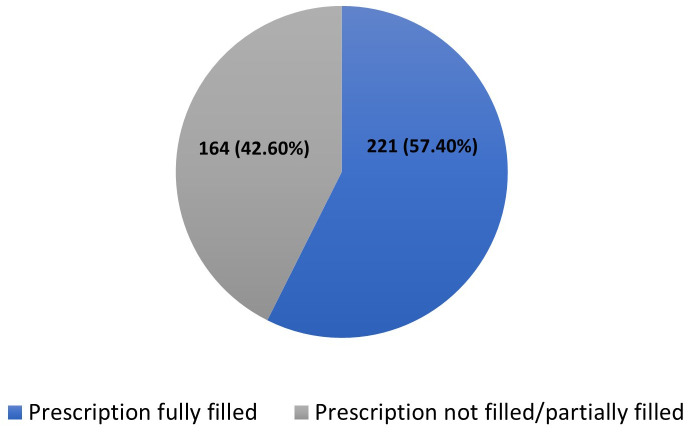
Proportion of prescription fulfillment.

Less than half of the participants; 164/385(42.60%), had their prescriptions fully filled (Fig 1).

The average prescription fulfillment rate was 66.36%. Antidepressants were observed to have the highest fulfillment rate of 95.14% (Table 2).

**Table 2:** Prescription fulfillment per drug.

Pharmacological class	Drug	Dispensed/Prescribed (%)
**Antipsychotics**	Stelazine	72/72 (100.00)
	Fluphenazine	62/64 (96.88)
	Chlorpromazine	70/77 (90.91)
	Haloperidol	17/21 (80.95)
	Flupenthixol	9/20 (45.00)
	Risperidone	13/36 (36.11)
	Quetiapine	4/15 (26.67)
	Zuclopenthixol	1/11 (9.09)
	Olanzapine	5/62 (8.06)
	**Average percentage**	**54.85**
**Antidepressants**	Amitriptyline	27/27 (100.00)
	Fluoxetine	65/72 (90.28)
	**Average percentage**	**95.14**
**Mood stabilizers/ Antiepileptics**	Diazepam	21/21 (100.00)
	Phenobarbital	6/6 (100.00)
	Clonazepam	2/2 (100.00)
	Carbamazepine	121/131 (92.37)
	Sodium valproate	66/89 (74.16)
	Lamotrigine	17/24 (70.83)
	Lithium	1/2 (50.00)
	Phenytoin	4/10 (40.00)
	**Average percentage**	**78.42**
**Anticholinergics**	Trihexyphenidyl	151/234 (64.52)
	Procyclidine	2/21 (9.52)
	**Average percentage**	**37.02**
**Overall Fulfillment rate**		**66.36%.**

### Patients who have experienced drug stock-outs

**Fig 2:**
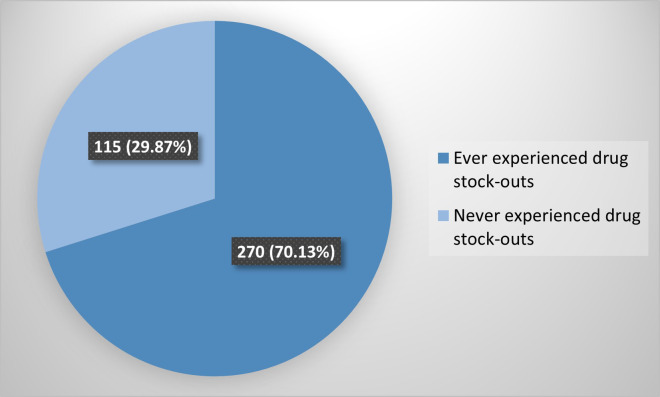
Prevalence of patients who have experienced drug stock-outs.

Majority of the patients experienced drug stock-outs; 270/385(70.13%) (Fig 2).

### Coping mechanisms of patients to drug stock-outs

Out of the 599 responses on coping mechanisms to drug stock-outs, the most popular were; out-of-pocket purchase of drugs from pharmacies; 195/599(32.55%), returning to hospital on a later date; 129/599(21.53%), skipping drug doses; 82/599(13.69%) and using unprescribed herbal remedies 54/599(9.02%) (Table 3).

**Table 3:** Coping mechanisms of participants.

Coping mechanisms	Frequency n = 599(%)
**Adaptive Mechanisms**	
Out-of-pocket purchase from pharmacies	195(32.55)
Return to hospital on another day	129(21.53)
Seek drugs from other public facilities	43(7.18)
Consult with healthcare providers for alternatives	16(2.67)
**Maladaptive Mechanisms**	
Skip doses	82(13.69)
Use of unprescribed herbal remedies	54(9.02)
Seek spiritual healing	30(5.01)
Seek traditional healing	22(3.67)
Get drugs from peers	19(3.17)
None, I do not have any other coping mechanisms	5(0.84)
Other coping mechanisms	4(0.67)

### Expenditure on drugs

**Fig 3:**
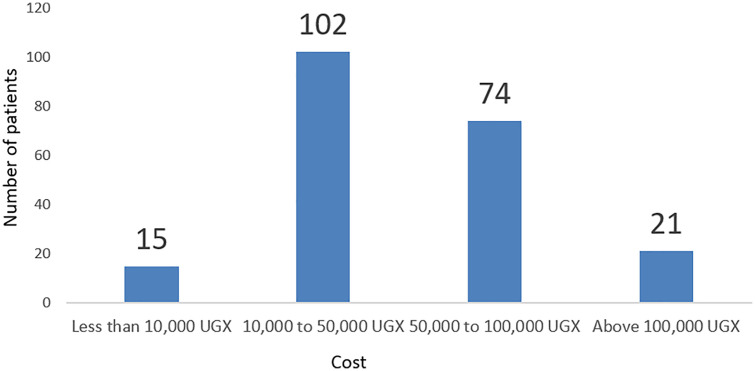
Expenditure on out-of-pocket purchase of drugs.

Majority of participants; 102/385(26.49%), spent between Uganda shillings 10,000–50,000 on out-of-pocket purchase of drugs (Fig 3).

### Undesirable outcomes of coping mechanisms to drug stock-outs

**Fig 4:**
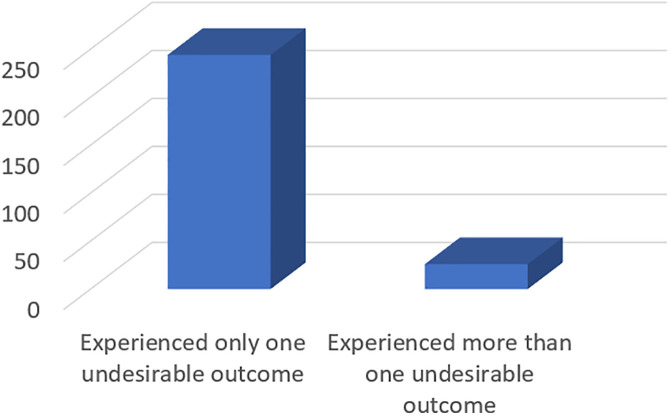
Distribution of participants based on number of undesirable outcomes experienced.

There were 197 reports of undesirable outcomes of coping mechanisms to drug stock-outs. The commonest were; worsening of symptoms; 43/197(21.8%), insomnia; 38/197(19.3%), and relapses; 32/197(16.2%) (Table 4). Out of 270 participants who were victims of drug stock-out, 26(9.63%) reported experiencing more than one undesirable outcome of their coping mechanism (Fig 4).

**Table 4:** Undesirable outcomes of coping mechanisms faced by respondents.

Undesirable outcomes	Frequency n = 197(%)
Worsening of symptoms	43 (21.8)
Insomnia	38 (19.3)
Symptoms reoccurred/relapsed	32 (16.2)
Pain	14 (7.1)
Reduced productivity	12 (6.0)
Increased expenses	12 (6.0)
Restlessness	11 (5.6)
Panic attacks	8 (4.0)
Increased stress	7 (3.6)
Depression	7 (3.6)
Body weakness	6 (3.0)
Increased anxiety	6 (3.0)
Were discriminated against	1 (0.8)

## Discussion

Prescription fulfillment rates were low in the current study. The proportion of patients whose prescriptions were fully filled was slightly less than half. These results were similar to those observed in a study conducted in Mumbai, India which reported that half of prescriptions were filled [27]. Furthermore, a survey from Twaweza in Tanzania showed that 41% of patients who had experienced drug stockouts at a public facility had resorted to procuring drugs from private health facilities [28]. The similarity across these studies may stem from the fact that they were all carried out in low-and middle-income countries (LMICs). Drug stockouts is a common challenge impacting public healthcare systems in LMIC economies [29]. It is important to note, however, that the research conducted in Tanzania and India considered general patient populations, while the current study at BNRMH specifically focused on mental health patients who were attending the outpatient department.

The low proportion of prescription fulfillment noted in this study and other comparable research could significantly affect the outcomes of patient treatment. Given the socioeconomic challenges in LMICs, patients often find difficulty purchasing prescribed drugs out-of-pocket [30]. This is because of the significant financial strain it causes to the affected patients [31]. Furthermore, inadequate access to drugs results in worsening of illnesses or incomplete recovery. Patients’ trust and confidence in the healthcare system is also lost when they experience frequent drug stock-outs [32]. Ensuring consistent availability of essential drugs is crucial for both patient prognosis and the overall public image of the healthcare system [29].

Patients at BNRMH employed a range of coping mechanisms to deal with unfilled prescriptions. Research on how persons living with mental health illnesses handle unmet prescription delivery is currently limited in Uganda. However, in the current study adaptive coping mechanisms included; obtaining drugs from other public medical facilities, returning to the hospital to seek drugs on a later day, purchasing prescribed drugs from private community pharmacies and seeking advice from health workers on alternative treatment options.

These coping mechanisms, in some cases, may have provided short-term solutions, however, they are not sustainable [33]. For instance, patients’ socioeconomic well-being may be strained if they must often pay for their drugs out-of-pocket. This is because mental diseases are chronic and require long-term drug treatment [34]. Consequently, numerous patients eventually skip doses of their prescribed drugs. Low health professional to patient ratios are a common problem in LMIC healthcare systems [35]. There is specifically an overall shortage of health workers with specialized training in mental healthcare [36]. The already overwhelmed healthcare system is further burdened if patients frequently need to return to the hospital to obtain drugs they previously did not get or to consult with another medical professional about alternative treatment options.

The participants’ maladaptive coping mechanisms examined in this study included using drugs obtained from peers, skipping doses, using unprescribed herbal drugs, consulting spiritual or traditional healers whereas some reported not having any coping mechanisms. In a research conducted in central Uganda in the districts of Mukono and Buikwe, similar coping mechanisms were noted among patients with diabetes and hypertension [22]. Shortage of drugs in Ugandan healthcare system has also been observed to affect care of other health conditions such as; HIV, TB, malaria, non-communicable diseases and even conditions affecting maternal and child healthcare [37–40]. This underpins the fact that drug stock-outs in Uganda is not unique to mental healthcare but is indeed a general healthcare problem [41]. In light of the knowledge gap on drug shortages in mental healthcare in Uganda, this study draws a comparison with findings from the treatment of other chronic conditions. This comparison is justifiable because often times some patients living with mental illnesses may have co-occurring illnesses whose treatment is also compromised by stock-out of drugs. In scenarios like this maladaptive coping mechanisms to drug stock-outs further compound poor outcomes of treatment and even jeopardize the patients’ overall safety [42].

In the current study participants reported some undesirable outcomes as a result of coping mechanisms. These included; worsening of symptoms, insomnia, relapse of symptoms and low productivity. Some also reported incurring higher medical costs. Due to drug stockouts these participants were compelled to miss their prescribed treatment. They therefore resorted to alternative treatment options to cope with the situation. However, poor adherence to prescribed treatment is associated with poor prognosis [43]. Therefore, it is not surprising that coping mechanisms such as; skipping treatment and using non-conventional treatment methods like spiritualism and traditional medicine could have led to these undesirable outcomes in the current study. African communities have practiced traditional medicine and spiritualism for millennia [44–46]. However, the scarcity of conventional scientific studies about the effectiveness, safety, and repeatability of these long-standing cultural practices has significantly made them unpopular in contemporary practice of medicine [47–49].

Persons living with mental illnesses can be supported to not only have access to drugs but also to improve their treatment outcomes. This entails improving access to healthcare services like; counseling programs where patients are seen by clinical psychologists who provide psychotherapy [50]. Forming community support networks where patients convene to share experiences and learn from each other helps to fight low self-esteem among mental health patients [51]. Training patients on positive living skills like relaxation techniques and productive leisure activities prevents patients from seeking harmful treatment alternatives [52]. Empowering health professionals with supply chain management skills improves inventory management of drugs and other supplies to reduce stock-outs [41]. This can be realized by eliminating bureaucratic processes in procurement of drugs and implementing effective supply chain policies [53]. For instance, in the context of Uganda, consumption of medical supplies is variable in various facilities. Therefore, the pull model of procurement would be desirable as opposed to the push model.

Furthermore, access to drugs can be enhanced by staffing the supply chain workforce with competent and skilled professionals [54], increasing financing towards the health supply chain [55] and optimizing the use of effective logistic management information systems [56]. Pharmacists are healthcare cadres that are specifically trained to handle supply chain systems of drugs. They play a significant role in improving inventory management of drugs [57,58]. Optimally utilizing their expertise in healthcare systems in sub-Saharan Africa would significantly improve patient access to pharmaceutical supplies.

## Conclusion

Prescription fulfillment rates were generally low in the current study due to frequent drug stock-outs at BNRMH outpatient department. This consequently led most patients to become poorly adherent to their prescribed treatment. Such patients were compelled to find alternative ways to acquire prescribed drugs. Those who could not afford to purchase prescribed drugs out-of-pocket skipped their treatment doses or resorted to treatment modalities considered unconventional within the healthcare system of Uganda. These patients who were victims of drug stock-outs consequently reported experiencing undesirable outcomes that could potentially jeopardize patient prognosis and safety. Pharmacists as drug experts could play a significant role in performing extensive research on complementary and alternative medicines to provide options to patients in cases of drug stock-outs. These remedies that include herbal medicines have been locally used for millennia and are accessible to the communities. With objective evidence of safety and efficacy they can be adopted as alternatives in cases where conventional treatment options are unavailable or ineffective.

### Limitations

The current study was conducted in BNRMH the national referral facility for mental healthcare in Uganda. The findings may not be generalizable to other mental health facilities within Uganda given there may be varying demand for drugs and other consumables depending on the facility. The current study being a cross-sectional study only offers data on a limited period of time and may not be generalizable to a longer period. Patients were interviewed to gather data for the current study. Recall and interviewer bias may have confounded the findings. Efforts were made to lessen this by cross-referencing some of the information gathered with the facility’s patient records. The current study also did not investigate the causes of the observed drug stock-outs.

## Supporting information

S1 File Dataset 1. (XLSX)

S2 File Dataset 2. (XLSX)

## Abbreviations

ADHD: Attention Deficit Hyperactivity Disorder
BNRMH: Butabika National Referral Mental Hospital
DALY: Disability Adjusted Life Year
HIV: Human Immunodeficiency Virus
LMIC: Low-and middle-income country
OPD: Outpatient department
TB: Tuberculosis
WHO: World Health Organization
YLD: Year Lived with Disability
